# Byways of Medical Relief

**Published:** 1911-10-21

**Authors:** 


### BYWAYS OF MEDICAL RELIEF.

(Contributions are invited to this column.)

### II.—Institutions which Help the Hospitals.

Experience has shown that it is wiser not to attempt on a very small scale the acute medical and surgical work of general hospitals. It is better in country localities or small towns to concentrate attention on the provision of a good district nurse and undertake the care of the sick in their own homes rather than to aim at running a little cottage hospital which is nearly empty half the year. And yet the pressure on the beds of most county infirmaries appears to justify efforts to provide local accommodation for the sick where means are forthcoming. How best can the genuine enthusiasm for relieving suffering exhibited in every part of the kingdom be turned to account without involving subscribers in the bottomless sea of hospital problems?

Modern methods of treatment and modern methods of locomotion are working together to supply the answer. It is instructive to study the average duration of each patient's stay in the various London and provincial hospitals. It varies from about twenty-one days at the London Hospital to twenty-eight at St. Bartholomew's; from eighteen days at the Newcastle Royal Infirmary and the Leeds General Infirmary to twenty-seven days at the Sheffield Royal Infirmary; from nineteen days at the Poplar Hospital for Accidents to thirty-nine at the Seamen's; from twenty at the Wolverhampton and the Coventry Hospitals to forty at the Royal Devon and Exeter Hospital. Allowing for some difference in the class of cases received, does it not appear evident that some hospitals enjoy greater facilities than others for emptying their beds into subsidiary institutions where the cure commenced in the hospital wards may be brought to a conclusion without danger to the patient? Every year is bringing closer home to the physician and surgeon the imperative need for getting patients out of the towns where the big hospitals are perforce located into purer air as soon as the crisis is over. Every year makes it easier to effect the transport of sick folk without exposing them to discomfort or danger. But the facilities for recovery in fresh air and suitable surroundings do not increase in proportion to the need. There is room for an indefinite expansion of convalescent aid through the means of the little hospitals scattered up and down the country, some of them vainly struggling to perform ambitious work, and failing to satisfy the aspirations of their most devoted supporters.

A little institution at Newbury affords a good illustration of the process of adaptation going on in work of this description. The Speen Cottage Hospital at Newbury was founded in 1869 in a singularly healthy small country town, within fifteen miles of the county hospital at Reading. It was equipped with five beds, and it continued to exist under the title of a cottage hospital till 1889. By that time experience had shown that it was less well suited for the reception of acute cases than for convalescents. Accordingly in that year the name was changed to Speen Convalescent Home, and it has continued a useful career ever since, and in the year 1909 relieved fifty-seven patients. It is greatly to be wished that this process of readjustment could be carried through wherever actual hospital work has proved beyond the resources of the little cottage hospitals, and this is the case in more institutions than have yet come to acknowledge facts and the relentless march of progress. But in order to prove of the greatest possible value in the chain of medical assistance, these small locally supported homes ought to be linked up with the hospitals and receive, at any rate, a proportion of their patients from this source. There can be no reasonable doubt that if this were done hospitals now detaining their patients twenty-eight days could pass them out of the wards in twenty-one, and thus make room for 25 per cent. more of additional sufferers. One of the great drawbacks to the extension of convalescent aid by the local homes to hospital patients lies in the ticket system. Subscribers want beds for their domestics or for the relatives of their domestics. Or they want to be assured that the beds are reserved for residents in the parish, and they look coldly on the needs of the work-people in the manufacturing towns in the county. All of which proves the need for educating them in larger principles of charity. It is to the county health societies we must look to develop the sense of corporate responsibility and widen the boundaries of altruism. But hospitals might do much to encourage the little institutions to co-operate with them by the use of the silver key. For a fee of from 5s. to 7s. 6d. a week they could procure admission for their out-going patients to many a little cottage hospital or local convalescent home which now languishes for lack of a constant supply of suitable applicants for admission. It would pay the supporters of such homes well to keep the beds always full by admitting hospital patients at a greatly reduced charge, for such little establishments cost very nearly as much when the beds are empty as when they are full. In return for the swift reception of patients whose cure was but half completed, the hospital would no doubt be willing to give a preference to cases from the locality in which the home is situated and the interchange would be of great mutual value.

Among the private institutions which shun publicity and are dependent almost entirely on the means and energy of one individual few are doing better work for the country than those which offer convalescent aid. Amongst them are the Skegness Home with sixteen beds, supported by Frederica Countess of Scarbrough and admirably managed by Miss Charles. In London there is Erskine House on Hampstead Heath, largely supported by the Guild of Compassion, and under the personal care of Canon and Mrs. Barnett. This little home is in touch with the London Hospital, from which a large proportion of the patients are received, and is inspired by the best instincts of personal devotion to the poor.

